# Documentation of body mass index and control of associated risk factors in a large primary care network

**DOI:** 10.1186/1472-6963-9-236

**Published:** 2009-12-16

**Authors:** Stephanie A Rose, Alexander Turchin, Richard W Grant, James B Meigs

**Affiliations:** 1 Division of General Internal Medicine, University of Kentucky, 740 South Limestone Street, Kentucky Clinic K507, Lexington, KY 40536, USA; 2 Clinical Informatics Research and Development, Harvard University, and Division of Endocrinology, Brigham and Women's Hospital, 221 Longwood Avenue, Boston, MA, 02115, USA; 3 General Medicine Division, Massachusetts General Hospital, 50 Staniford Street, Boston, MA, 02114, USA; 4 General Medicine Division, Massachusetts General Hospital, 50 Staniford Street, Boston, MA, 02114, USA

## Abstract

**Background:**

Body mass index (BMI) will be a reportable health measure in the United States (US) through implementation of Healthcare Effectiveness Data and Information Set (HEDIS) guidelines. We evaluated current documentation of BMI, and documentation and control of associated risk factors by BMI category, based on electronic health records from a 12-clinic primary care network.

**Methods:**

We conducted a cross-sectional analysis of 79,947 active network patients greater than 18 years of age seen between 7/05 - 12/06. We defined BMI category as normal weight (NW, 18-24.9 kg/m^2^), overweight (OW, 25-29.9), and obese (OB, ≥ 30). We measured documentation (yes/no) and control (above/below) of the following three risk factors: blood pressure (BP) ≤130/≤85 mmHg, low-density lipoprotein (LDL) ≤130 mg/dL (3.367 mmol/L), and fasting glucose <100 mg/dL (5.55 mmol/L) or casual glucose <200 mg/dL (11.1 mmol/L).

**Results:**

BMI was documented in 48,376 patients (61%, range 34-94%), distributed as 30% OB, 34% OW, and 36% NW. Documentation of all three risk factors was higher in obesity (OB = 58%, OW = 54%, NW = 41%, p for trend <0.0001), but control of all three was lower (OB = 44%, OW = 49%, NW = 62%, p = 0.0001). The presence of cardiovascular disease (CVD) or diabetes modified some associations with obesity, and OB patients with CVD or diabetes had low rates of control of all three risk factors (CVD: OB = 49%, OW = 50%, NW = 56%; diabetes: OB = 42%, OW = 47%, NW = 48%, p < 0.0001 for adiposity-CVD or diabetes interaction).

**Conclusions:**

In a large primary care network BMI documentation has been incomplete and for patients with BMI measured, risk factor control has been poorer in obese patients compared with NW, even in those with obesity and CVD or diabetes. Better knowledge of BMI could provide an opportunity for improved quality in obesity care.

## Background

Obesity is a common problem in the United States that independently confers risk for chronic disease and early mortality [1]. Several studies address the importance of recognition and treatment of obesity in chronic disease management, but few evaluate body mass index (BMI) assessment and risk factor documentation and control in patients who are obese both with and without cardiovascular disease (CVD), diabetes or risk factors [2-6].

Assessments of BMI and CVD or diabetes risk factors are simple, inexpensive tools that can be used by primary care physicians (PCPs) to address obesity and its complications. Little consensus exists in current guidelines concerning the need to screen for BMI and associated risk factors [7-15]. In 1998 the National Heart, Lung, and Blood Institute (NHLBI) established guidelines for BMI and risk factor measurement in all patients under 80 years of age with the goal of implementation of strategies for weight loss and risk factor control in patients with a BMI of ≥ 30 kg/m^2 ^or 25-29.9 kg/m^2 ^and ≥ two risk factors [10]. In 2007 the National Committee for Quality Assurance (NCQA) organized field tests of a prospective Healthcare Effectiveness Data and Information Set (HEDIS) measure to assess the percentage of members 18-74 years of age with an outpatient visit, and who had BMI documented and risk factors documented if found to have a BMI of ≥30 kg/m^2 ^[16]. Their work led to the proposed HEDIS 2009 measure *BMI Assessment *[17], which is proposed for reporting by commercial, Medicaid, and Medicare plans.

Given the magnitude and clinical impact of the current obesity epidemic and its risk factors, and the potential implementation of the HEDIS *BMI Assessment *measure, we aimed to examine current care in BMI and risk factor assessment for patients in a large network of primary care practices that use a common electronic health record (EHR). We investigated the prevalence of BMI measurement and documentation and control of the CVD and diabetes risk factors blood pressure (BP), low density lipoprotein (LDL), and fasting and casual glucose. We examined these risk factors according to the BMI categories normal weight, overweight, and obese. We hypothesized that documentation of BMI would vary widely in clinical practice, and that patients who were obese would be more likely to have better documentation, but poorer control, of risk factors than patients of normal weight.

## Methods

### Setting and Patients

We identified 138,933 patients in our primary care research network of twelve practices that make up the Massachusetts General Hospital Primary Care Practice Based Research Network (PBRN). Practices include urban academic faculty practices, private offices, and community health centers in and around Boston, Massachusetts. These twelve practices use a common EHR that contains all clinical and utilization data for each patient. The data from the EHR data are searchable in the Research Patient Data Repository (RPDR) [18]. From these, we included 79,947 patients who were at least 18 years of age and had at least two clinic visits billed to their PCP between 7/1/05 and 12/31/06, and did not have a height greater than or equal to seven feet (2.13 meters), weight <70 or >1000 pounds (<31.8 or >453.6 kilograms), systolic BP <50 or >260 mmHg, or diastolic BP <30 or >150 mmHg. The study was approved by the Partners Health Care System Institutional Review Board.

### BMI Measurement

The intent of the HEDIS initiative is to measure and increase documentation of BMI. We obtained BMI from height and weight data recorded in the EHR, where they are used to automatically calculate and display BMI. For this analysis we calculated BMI from the most recent weight in the 18-month period and the most recent height prior to 12/31/06 from structured coded entries in the EHR. For completeness, we also searched the text of narrative notes in the EHR using a validated Natural Language Processing abstraction tool that computationally abstracts weight, height, and BP values from the free text of clinician notes [19,20]. The sensitivity and specificity of the approach to abstraction (compared with a trained chart abstractor) are 87.9% and 99% for detection of height, 91.8% and 92.1% for detection of weight, and 91% and 96% for detection of BP [19,20]. Because of high rates of missing heights, we confirmed with clinic site medical directors that we had searched in all the appropriate places in the clincial record for height and weight information.

We categorized BMI using Centers for Disease Control (CDC) definitions, with underweight equal to BMI <18.5 kg/m^2^, normal weight equal to a BMI of 18.5-<25 kg/m^2^, overweight BMI equal to 25-<30 kg/m^2^, and obese BMI ≤30 kg/m^2 ^[21]. We included underweight patients when measuring documentation of BMI, but excluded them otherwise. Among those with a documented BMI, we examined documentation and control of three risk factors: blood pressure (BP), LDL cholesterol, and fasting or casual glucose levels, or both. We examined risk factor documentation and control in the clinic population overall, and stratified by three BMI categories.

### Risk Factor, CVD, and Diabetes Measurement

Documentation of a risk factor was defined as being found in the EHR at least once within the study period. If a risk factor was documented more than once, we used the most recently documented value. A risk factor was defined as controlled if within normal range, defined as: BP ≤130/≤85 mmHg, LDL<130 mg/dL (3.367 mmol/L), and fasting glucose <100 mg/dL (5.55 mmol/L) or casual glucose <200 mg/dL (11.1 mmol/L) [22-24]. We also measured documentation and control of all three risk factors combined as an aggregate measure of risk factor management.

We defined clinical CVD as coronary artery disease, cerebrovascular accident, or peripheral vascular disease listed on the EHR Problem List, or as having two outpatient or one inpatient International Classification of Diseases, Ninth Revision (ICD-9) codes for CVD. We defined clinical diabetes as diabetes on the EHR problem list and a diabetes medication on the medication list, or as having two outpatient or one inpatient codes related to diabetes. The definitions of diabetes, hypertension, hyperlipidemia and CVD have previously been validated [25,26]. For instance, the sensitivity of our approach for diabetes or CVD are >98% and specificity of >97% compared to the gold standard of trained research nurse chart abstraction. We used the same approach as for diabetes to define hypertension and hyperlipidemia. Other measurements included age, race, gender, number of PCP visits during the study period, insurance type, and clinic site.

### Statistical Analysis

We conducted a cross-sectional analysis. We measured rates of BMI documentation in each clinic site and overall. Among those with a BMI, we measured risk factor documentation and control overall, and stratified by the three BMI categories. We further stratified the analysis by the presence or absence of CVD or diabetes. We used generalized linear models or chi-square tests to test levels or rates of characteristics by BMI category. We used the Breslow Day test for homogeneity of odds ratios to test for effect modification of CVD or diabetes on the association of BMI with risk factor documentation and control. We used SAS version 9.1 (Cary, NC) for all analyses, and considered a p value of <0.05 to indicate statistical significance.

## Results

### BMI Documentation and Characteristics of Patients with Documented BMI

Of 79,947 patients, 72,633 (90.9%) had an available weight, 50,345 (63.0%) had an available height, and 48,376 (60.5%) had both height and weight recorded to calculate BMI (Table 1). Of those with BMI present, 14,290 patients (30%) were classified as obese, 16,402 (34%) overweight, 16,936 (36%) normal weight, and 748 (2%) underweight. Compared to those missing BMI, patients with BMI documented were younger, had a higher mean number of PCP visits, were more likely to be women, and were more likely to have commercial insurance or Medicare than patients without a BMI (p < 0.0001 for all; Table 2). As expected, patients with obesity were older, less likely to be women, white, or to have private insurance or Medicare, had a higher mean number of PCP visits, and were more likely to have a history of CVD, diabetes, hypertension, and dyslipidemia than patients of normal weight (p < 0.0001 for all; Table 3).

**Table 1:** Body Mass Index Documentation in 79,947 Patients in 12 Primary Care Clinics

Clinic	N	BMI	Height Documented	Weight Documented
		**%**	**%**	**%**
**1**	**3,027**	93.5	94.1	99.1
**2**	**4,559**	89.8	95.7	91.6
**3**	**3,274**	86.9	87.5	99.1
**4**	**3,989**	85.8	94.5	87.4
**5**	**1,344**	75.1	75.2	99.5
**6**	**7,747**	65.8	66.0	99.0
**7**	**21,299**	64.0	65.8	96.1
**8**	**3,962**	55.4	55.8	98.7
**9**	**12,919**	50.2	55.4	71.1
**10**	**4,781**	41.9	44.4	78.4
**11**	**8,052**	38.2	38.3	99.0
**12**	**4,994**	34.0	35.7	88.9
***Total***	***48,376***	***60.5***	***63.0***	***90.9***

Data are ranked by BMI documentation rate, include patients at least 18 years of age with at least two clinic visits billed to their PCP between 7/1/05 and 12/31/06, and include underweight patients (BMI <18.5 kg/m^2^) excluded from the main analysis. BMI = Body Mass Index (weight in kilograms/height in meters^2^)

**Table 2:** Patient Demographics and Clinical Characteristics in 79,947 Patients in 12 Primary Care Clinics by Documentation or Missingness of BMI

Characteristic			BMI Documented	48,376	BMI not Documented	31,571	P value
**Clinic**	**n**	**%**					**<0.0001**
**1**			2,830	5.9	197	0.6	
**2**			4,093	8.5	466	1.5	
**3**			2,844	5.9	430	1.4	
**4**			3,421	7.1	568	1.8	
**5**			1,009	2.1	335	1.1	
**6**			5,095	10.5	2,652	8.4	
**7**			13,630	28.2	7,669	24.3	
**8**			2,194	4.5	1,768	5.6	
**9**			6,489	13.4	6,431	20.4	
**10**			2,003	4.1	2,778	8.8	
**11**			3,073	6.4	4,979	15.8	
**12**			1,696	3.5	3,298	10.5	
**Age**	**mean years**		51		53		<0.0001
**Women**	n	%	31,412	64.9	15,986	50.6	<0.0001
**Race**	n	%					<0.0001
**White**			37,625	77.8	25,264	80.0	
**Asian**			2,057	4.3	1,426	4.5	
**Black**			2,908	6.0	1,464	4.6	
**Hispanic**			4,123	8.5	2,397	7.6	
**Number of PCP visits**	**mean**	**range**	4.2	(2-61)	3.9	(2-56)	<0.0001
**Commercial Insurance or Medicare**	**n**	**%**	42,740	88.4	27,522	87.2	<0.0001
**History of CVD**	**n**	**%**	7,298	15.1	4,989	15.8	0.0060
**History of diabetes**	**n**	**%**	5,872	12.1	3,453	10.9	<0.0001
**History of hypertension**	**n**	**%**	22,121	45.7	14,698	46.6	0.02
**History of dyslipidemia**	**n**	**%**	25,698	53.1	17,133	54.3	0.002
**HbA1c**	**%**		6.7		6.8		0.0001
**Blood pressure**	**mean mmHg**		122/75		123/74		<0.0001
**Total cholesterol**	mean mg/dL (mmol/L)		188.1 (4.87)		188.5 (4.88)		0.29
**LDL**	mean mg/dL (mmol/L)		104.1 (2.70)		105 (2.72)		0.001
**HDL**	mean mg/dL (mmol/L)		59.3 (1.54)		57.8 (1.50)		<0.0001
**Triglyceride level**	mean mg/dL (mmol/L)		127.8 (1.44)		132.6 (1.50)		<0.0001
**Casual glucose**	mean mg/dL (mmol/L)		101.8 (5.65)		100.8 (5.59)		0.0007
**Fasting glucose**	mean mg/dL (mmol/L)		102.4 (5.68)		100.9 (5.60)		0.0007

**Risk factor documentation**	**Total n**	**%**					

**Blood Pressure**	64,204	80.3	37,489	77.5	26,715	84.6	<0.0001
**LDL**	55,210	69.1	33,788	69.8	21,422	67.9	<0.0001
**Casual glucose**	56,984	71.3	35,125	72.6	21,859	69.2	<0.0001
**Fasting glucose**	21,533	26.9	13,533	28.0	8,000	25.3	<0.0001
**HbA1c**	14,252	17.8	9,367	19.4	4,885	15.5	<0.0001
**Total cholesterol**	58,951	73.7	36,247	74.9	22,704	71.9	<0.0001
**HDL**	58,373	73.0	35,874	74.2	22,499	71.3	<0.0001
**Triglycerides**	55,948	70.0	34,247	70.8	21,701	68.7	<0.0001

**Risk factor control in those with documented risk factor**	**Total n**	**%**					

**Blood Pressure <130 and < 85 mmHg**	46,748	72.8	27,212	72.6	19,536	73.1	0.13
**LDL <130 mg/dL**	43,369	78.6	26,656	78.9	16,713	78.0	0.015
**Casual glucose < 200 mg/dL**	55,642	97.6	34,231	97.5	21,411	98.0	0.0001
**Fasting glucose < 100 mg/dL**	13,782	64.0	8,574	63.4	5,208	65.1	0.01
**HbA1c <7%**	9,638	67.6	6,463	69.0	3,175	65.0	<0.0001
**Total cholesterol <200 mg/dL**	37,279	63.2	22,952	63.3	14,327	63.1	0.59
**HDL >35 mg/dL (male) and >40 mg/dL (female)**	52,971	90.8	32,742	91.3	20,229	89.9	<0.0001
**Triglycerides <150 mg/dL**	48,188	86.1	29,675	86.7	18,513	85.3	<0.0001

P-value compares BMI documented with BMI not documented. BMI = Body Mass Index (weight in kilograms/height in meters^2^), PCP = Primary Care Physician, CVD = Cardiovascular Disease, HbA1c = Hemoglobin A1C, LDL=low density lipoprotein cholesterol, HDL = high density lipoprotein cholesterol. LDL <130 mg/dL = <3.367 mmol/L; casual glucose <200 mg/dL = <5.55 mmol/L; fasting glucose <100 mg/dL = <11.1 mmol/L

**Table 3:** Characteristics of 47,628 Primary Care Patients by BMI Category

Characteristic		Obese	n = 14,290	Overweight	n = 16,402	Normal	n = 16,936	P value
**Age**	**(mean years)**	53		53		48		<0.0001
**Number of PCP visits**	**(mean)**	4.7		4.2		3.9		<0.0001
**Women**	**n (%)**	8,565	59.9	8,899	54.3	13,272	78.4	<0.0001
Race*	**n (%)**							<0.0001
**White**		10,712	75.0	12,674	77.3	13,655	80.6	
**Asian**		179	1.3	567	3.5	1,229	7.3	
**Black**		1,244	8.7	989	6.0	649	3.8	
**Hispanic**		1,722	12.1	1,561	9.5	811	4.8	
**Private Insurance or Medicare**	**n (%)**	12,104	84.7	14,502	88.4	15,467	91.3	<0.0001
**History of CVD**	**n (%)**	2,647	18.5	2,699	16.5	1,862	11.0	<0.0001
**History of diabetes**	**n (%)**	3,238	22.7	1,789	10.9	824	4.9	<0.0001
**History of hypertension**	**n (%)**	9,116	63.8	7,895	48.1	4,931	29.1	<0.0001
**History of dyslipidemia**	**n (%)**	9,235	64.6	9,495	57.9	6,769	40.0	<0.0001

P-value is for general association across BMI categories. BMI = Body Mass Index (weight in kilograms/height in meters^2^), PCP = Primary Care Physician, and CVD = Cardiovascular Disease. *Designation "Other" for race deleted from set.

### Documentation and Control of Risk Factors

**Figure 1:**
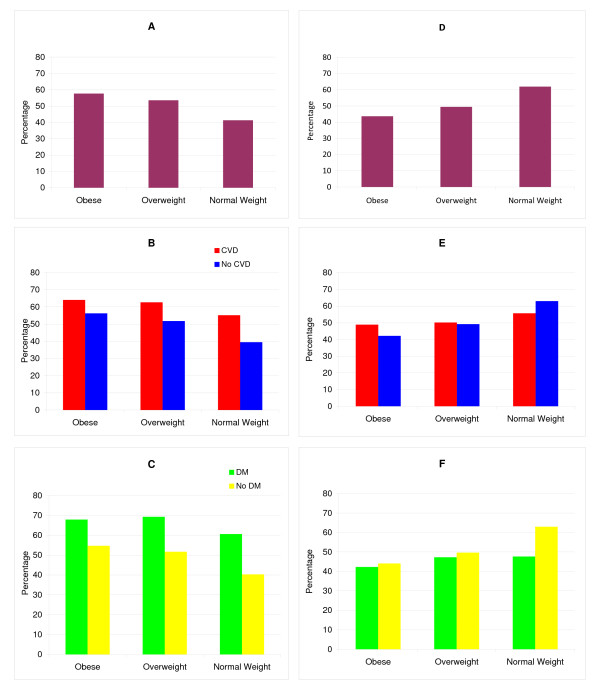
**Documentation and control of all three risk factors by BMI category**. A. Documentation of all 3 risk factors by BMI category; B. Documentation of all 3 risk factors by BMI category, stratified by history of CVD; C. Documentation of all 3 risk factors by BMI category, stratified by history of diabetes. D. Control of all 3 risk factors by BMI category; E. Control of all 3 risk factors by BMI category, stratified by history of CVD; F. Control of all 3 risk factors by BMI category, stratified by history of diabetes.

Overall, less than 78% had at least one risk factor documented and just half had all three documented (Table 4, Figure 1). Of these, about half of all patients had all three risk factors controlled (Figure 1). Documentation of all three risk factors was higher in obesity than in overweight or normal weight (p < 0.0001), but control of all three risk factors was lower, (p < 0.0001), with only 44% of patients with obesity having all risk factors under control.

**Table 4:** Documentation and Control of Risk Factors among 47,628 Primary Care Patients, by BMI Category

Risk Factor Documentation	Total	n = 47,628	Obese	n = 14,290	Overweight	n = 16,402	Normal	n = 16,936	P value
	**n**	**%**	**n**	**%**	**n**	**%**	**n**	**%**	

**BP**	36,934	77.6	10,900	76.3	12,890	78.6	13,144	77.6	<0.0001
**LDL**	33,427	70.2	11,239	78.7	12,013	73.2	10,175	60.1	<0.0001
**Casual Glucose**	34,589	72.6	11,216	78.5	11,901	72.6	11,472	67.7	<0.0001
**Fasting Glucose**	13,421	28.2	5,347	37.4	4,804	29.3	3,270	19.3	<0.0001

**Risk Factor Control**	**Total**		**Obese**		**Overweight**		**Normal**		**P value**

	**n**	**%**	**n**	**%**	**n**	**%**	**n**	**%**	

**BP <130 and < 85 mmHg**	26,736	72.4	6,801	62.4	9,202	71.4	10,733	81.7	<0.0001
**LDL <130 mg/dL**	26,328	78.8	8,777	78.1	9,273	77.2	8,278	81.4	<0.0001
**Casual glucose < 200 mg/dL**	33,698	97.4	10,707	95.5	11,635	97.8	11,356	99.0	<0.0001
**Fasting glucose < 100 mg/dL**	8,482	63.2	2,753	51.5	3,146	65.5	2,583	79.0	<0.0001

P-value is for general association across BMI categories. BMI=Body Mass Index (weight in kilograms/height in meters²); LDL <130 mg/dL=<3.367 mmol/L; casual glucose <200 mg/dL=<5.55 mmol/L; fasting glucose <100 mg/dL=<11.1 mmol/L.

The presence of CVD or diabetes modified some associations of BMI category with risk factor documentation and control. In patients with CVD or diabetes, the rates of documentation were generally less different comparing patients with obesity with those of normal weight, while in patients without CVD or diabetes, rates of documentation were strikingly higher in patients with obesity than normal weight (Figure 1, Table 5, p = 0.26 to <0.0001 for CVD- or diabetes-by-BMI category interaction). Likewise, in patients with CVD or diabetes the rates of control were generally less different comparing patients with obesity with those with normal weight, but in patients without CVD or diabetes, rates of control were strikingly lower in patients with obesity than normal weight (Figure 1, Table 6). Overall 50% or fewer of the patients with obesity and CVD or diabetes had all three risk factors under control (Figure 1, Tables 5 and 6).

**Table 5:** Documentation of Risk Factors in 47,628 Patients in 12 Primary Care Clinics Stratified by BMI Category and History of CVD or Diabetes

Risk Factors	Total		Obese	n = 14,290	Overweight	n = 16,402	Normal Weight	n = 16,936	P value

**History of CVD**	**n**	**%**	**n**	**%**	**n**	**%**	**n**	**%**	
**H/O CVD n = 7,208**			**H/O CVD N = 2,647**		**H/O CVD N = 2,699**		**H/O CVD N = 1,862**		
**NO H/O CVD n = 40,420**			**No H/O CVD N = 11,643**		**NO H/O CVD N = 13,703**		**NO H/O CVD N = 15,074**		


**BP**			**n = 10,900**		**n = 12,890**		**n = 13,144**		
**H/O CVD**	5,297	73.5	1,931	73.0	1,991	73.8	1,375	73.9	0.26
**NO H/O CVD**	31,637	78.3	8,969	77.0	10,899	79.5	11,769	78.1	
**LDL**			**n = 11,239**		**n = 12,013**		**n = 10,175**		
**H/O CVD**	6,199	86.0	2,393	90.4	2,358	87.4	1,448	77.8	0.15
**NO H/O CVD**	27,228	67.4	8,846	76.0	9,655	70.5	8,727	57.9	
**Casual glucose**			**n = 11,216**		**n = 11,901**		**n = 11,472**		
**H/O CVD**	6,305	87.5	2,355	89.0	2,322	86.0	1,628	87.4	0.0001
**NO H/O CVD**	28,284	70.0	8,861	76.1	9,579	69.9	9,844	65.3	
**Fasting glucose**			**n = 5,347**		**n = 4,804**		**n = 3,270**		
**H/O CVD**	2,741	38.0	1,158	43.8	1,023	37.9	560	30.1	<0.0001
**NO H/O CVD**	10,680	26.4	4,189	36.0	3,781	27.6	2,710	18.0	
**All 3 risk factors**			**n = 8,247**		**n = 8,797**		**n = 6,986**		
**H/O CVD**	4,417	61.3	1,696	64.1	1,693	62.7	1,028	55.2	<0.0001
**NO H/O CVD**	19,613	48.5	6,551	56.3	7,104	51.8	5,958	39.5	


**History of DM**			n	%	n	%	n	%	
**H/O diabetes n = 5,851**			**H/O diabetes N = 3,238**		**H/O diabetes N = 1,789**		**H/O diabetes N = 824**		
**NO H/O diabetes** **n = 41,777**			**NO H/O diabetes N = 11,052**		**NO H/O diabetes N = 14,613**		**NO H/O diabetes N = 16,112**		


**BP**			**n = 10,900**		**n = 12,890**		**n = 13,144**		
**H/O diabetes**	4,398	75.2	2,447	75.6	1,366	76.4	585	71.0	0.002
**NO H/O diabetes**	32,536	77.9	8,453	76.5	11,524	78.9	12,559	78.0	
**LDL**			**n = 11,239**		**n = 12,013**		**n = 10,175**		
**H/O diabetes**	5,285	90.3	2,963	91.5	1,617	90.4	705	86.2	0.51
**NO H/O diabetes**	28,142	67.4	8,276	74.9	10,396	71.1	9,470	58.8	
**Casual glucose**			**n = 11,216**		**n = 11,901**		**n = 11,472**		
**H/O diabetes**	5,225	89.3	2,884	89.1	1,596	89.2	745	90.4	<0.0001
**NO H/O diabetes**	29,634	70.9	8,332	75.4	10,305	70.5	10,727	66.6	
									
**Fasting glucose**			**n = 5,347**		**n = 4,804**		**n = 3,270**		
**H/O diabetes**	2,736	46.8	1,619	50.0	812	45.4	305	37.0	0.003
**NO H/O diabetes**	10,685	25.6	3,728	33.7	3,992	27.3	2,965	18.4	
**All 3 risk factors**			**n = 8,247**		**n = 8,797**		**n = 6,986**		
**H/O diabetes**	3,936	67.3	2,197	67.9	1,240	69.3	499	60.6	0.001
**NO H/O diabetes**	20,094	48.1	6,050	54.7	7,557	51.7	6,487	40.3	

P-value compares homogeneity of odds ratios across groups. CVD = Cardiovascular Disease, BMI = Body Mass Index (weight in kilograms/height in meters^2^), BP = Blood Pressure, LDL = low density lipoprotein cholesterol, HDL = high density lipoprotein cholesterol. All 3 risk factors documented = documentation of BP + LDL + casual OR fasting glucose. Numbers and percentages in table reflect percentage of risk factor documented by BMI type in patients either with or without a history of CVD or DM (i.e., 1,931 = 73% of 2,647 patients who are obese with BP documented with a history of CVD).

**Table 6:** Control of Risk Factors in 47,628 Patients in 12 Primary Care Clinics Stratified by BMI Category and History of CVD or Diabetes

Outcome	Total Documented		Total Controlled		Obese		Overweight		Normal		P value
**History of CVD**	**n**	**%**	**n**	**%**	**n**	**%**	**n**	**%**	**n**	**%**	
H/O CVD n = 7,208											
NO H/O CVD n = 40,420											

BP < 130 and < 85 mmHg					H/O CVD N = 1,931		H/O CVD N = 1,991		H/O CVD N = 1,375		
					NO H/O CVD 8,969		NO H/O CVD N = 10,899		NO H/O CVD N = 11,769		
						
					n = 6,801		n = 9,202		n = 10,733		
H/O CVD	5,297	73.5	3,338	63.0	1,155	59.8	1,256	63.1	927	67.4	<0.0001
NO H/O CVD	31,637	78.3	23,399	74.0	5,647	63.0	7,946	72.9	9,806	83.3	

LDL < 130 md/dL					H/O CVD N = 2,393		H/O CVD N = 2,358		H/O CVD N = 1,448		
					NO H/O CVD N = 8,846		NO H/O CVD N = 9,655		NO H/O CVD N = 6,989		
						
					n = 8,777		n = 9,273		n = 8,278		
H/O CVD	6,199	86.0	5,515	89.0	2,152	89.9	2,074	88.0	1,289	89.0	0.002
NO H/O CVD	27,228	67.4	20,813	76.4	6,625	74.9	7,199	74.6	6,989	80.1	

Casual glucose < 200 mg/dL					H/O CVD N = 2,355		H/O CVD N = 2,322		H/O CVD N = 1,628		
					NO H/O CVD N = 8,861		NO H/O CVD N = 9,579		NO H/O CVD N = 9,844		
						
					n = 10,707		n = 11,635		n = 11,356		
H/O CVD	6,305	87.5	5,979	94.8	2,173	92.3	2,218	95.5	1,588	97.5	0.13
NO H/O CVD	28,284	70.0	27,719	98.0	8,534	96.3	9,417	98.3	9,768	99.2	

Fasting glucose < 100 mg/dL					H/O CVD N = 1,158		H/O CVD N = 1,023		H/O CVD N = 560		
					NO H/O CVD N = 4,189		NO H/O CVD N = 3,781		NO H/O CVD N = 2,710		
						
					n = 2,753		n = 3,146		n = 2,583		
H/O CVD	2,741	38.0	1,325	48.3	415	35.8	561	54.8	349	62.3	0.0005
NO H/O CVD	10,680	26.4	7,157	67.0	2,338	55.8	2,585	68.4	2,234	82.4	

All 3 risk factors					H/O CVD N = 829		H/O CVD N = 1,693		H/O CVD N = 1,028		
					NO H/O CVD N = 2,767		NO H/O CVD N = 7,104		NO H/O CVD N = 5,958		
						
					n = 3,596		n = 4,344		n = 4,327		
H/O CVD	4,417	61.3	2,252	51.0	829	48.9	850	50.2	573	55.7	<0.0001
NO H/O CVD	19,613	48.5	10,015	51.1	2,767	42.2	3,494	49.2	3,754	63.0	


History of diabetes											
H/O diabetes n = 5,851											
NO H/O diabetes n = 41,777											

BP < 130 and < 85 mmHg					H/O diabetes N = 2,447		H/O diabetes N = 1,366		H/O diabetes N = 585		
					NO H/O diabetes 8,453		NO H/O diabetes N = 11,524		NO H/O diabetes N = 12,559		
						
					n = 6,801		n = 9,202		n = 10,733		
H/O diabetes	4,398	75.2	2,775	63.1	1,476	60.3	919	67.3	380	65.0	<0.0001
NO H/O diabetes	32,536	77.9	23,961	73.6	5,325	63.0	8,283	71.9	10,353	82	

LDL < 130 md/dL					H/O diabetes N = 2,963		H/O diabetes N = 1,617		H/O diabetes N = 705		
					NO H/O diabetes N = 8,276		NO H/O diabetes N = 10,396		NO H/O diabetes N = 9,470		
						
					n = 8,777		n = 9,273		n = 8,278		
H/O diabetes	5,285	90.3	4,746	89.8	2,635	88.9	1,462	90.4	649	92.1	0.57
NO H/O diabetes	28,142	67.4	21,583	76.7	6,143	74.2	7,811	75.1	7,629	80.6	

Casual glucose < 200 mg/dL					H/O diabetes N = 2,884		H/O diabetes N = 1,596		H/O diabetes N = 745		
					NO H/O diabetes N = 8,332		NO H/O diabetes N = 10,305		NO H/O diabetes N = 10,727		
						
					n = 10,707		n = 11,635		n = 11,356		
H/O diabetes	5,225	89.3	4,383	83.9	2,400	83.2	1,342	84.1	641	86.0	0.015
NO H/O diabetes	29,634	70.9	29,315	98.9	8,307	99.7	10,293	99.9	10,715	99.9	

Fasting glucose < 100 mg/dL					H/O diabetes N = 1,619		H/O diabetes N = 812		H/O diabetes N = 305		0.24
					NO H/O diabetes N = 3,728		NO H/O diabetes N = 3,992		NO H/O diabetes 2,965		
					n = 2,753		n = 3,146		n = 2,583		
H/O diabetes	2,736	46.8	554	20.2	285	17.6	170	20.9	99	32.5	
NO H/O diabetes	10,685	25.6	7,928	74.2	2,468	66.2	2,976	74.6	2,484	83.8	

All 3 risk factors					H/O diabetes N = 2,197		H/O diabetes N = 1,240		H/O diabetes N = 499		<0.0001
					NO H/O diabetes N = 6,050		NO H/O diabetes N = 7,557		NO H/O diabetes N = 6,487		
						
					n = 3,596		n = 4,344		n = 4,327		
H/O diabetes	3,936	67.3	1,754	44.6	929	42.3	587	47.3	238	47.7	
NO H/O diabetes	20,094	48.1	10,513	52.3	2,667	44.1	3,757	49.7	4,089	63.0	

P-value compares homogeneity of the odds ratios across groups. CVD = Cardiovascular Disease, BMI = Body Mass Index (weight in kilograms/height in meters^2^), BP = Blood Pressure, LDL = Low density lipoprotein cholesterol, HDL = High density lipoprotein cholesterol. Risk factor control rates are from patients with risk factors documented. Numbers and percentages in table reflect percentage of risk factor documented by BMI type in patients either with or without a history of CVD or DM (i.e., 1,931 = 73% of 2,647 patients who are obese with BP documented with a history of CVD). All 3 risk factors controlled = control of BP + LDL + casual OR fasting glucose. Documentation or control of all 3 risk factors defined as BP, LDL, and fasting or casual glucose below the thresholds indicated.

## Discussion

We examined the current state of BMI documentation and documentation and control of associated risk factors by BMI category, based on EHR data from almost 80,000 adult patients seen in a 12-clinic primary care network during 18 months in 2005-2006. Our findings demonstrate variations in risk factor recognition and control for obese persons compared to those of normal weight, as well as for those with and without CVD or type 2 diabetes. We found that documentation of BMI varied widely by clinic site and was overall low. Among patients with a BMI recorded, documentation of most risk factors was higher in patients with obesity compared with normal weight patients; however, control of risk factors was poorer in obesity than normal weight. Patients without a documented history of CVD or diabetes had strikingly more dissimilar rates of documentation and control between weight categories than patients with CVD or diabetes. Overall, patients with obesity with or without CVD or diabetes had lower rates of risk factor documentation and control than may be ideal given their high absolute risk of adverse health outcomes.

Our study builds upon findings from previous studies. Lemay *et al *audited medical records from 465 adult patients seen at a community health center during one week in February of 1999 to evaluate height and weight documentation and obesity diagnosis by the practitioner over a prior six-month period, and found that only 63% of their patient cohort had a height and weight documented [27]. Similar to our study, Lemay looked a group of patients in normal clinical practice; however, we were able to expand upon this study in terms of a far larger study sample as well as a longer sampling frame to assess provision of services (18 months versus 6 months), potentially providing a more accurate picture of risk factor evaluation. Six years after this study and seven years after the establishment of the NHLBI guidelines, we found the percentage of BMI documentation to be essentially the same (63% versus 60.5%). Rifas-Shiman *et al *studied 5,025 members of the same insurance plan and group practice who were a subset of participants from a cohort study and were continuously enrolled since 1999, had a visit in 2000, had a BMI measurement between January 1, 2000 and December 31, 2000, and who did not have medical conditions related to weight loss or CVD. They found that higher BMI was an independent predictor of increased fasting glucose, triglyceride, LDL cholesterol, and HDL cholesterol screening [28]. Rifas-Shiman identified lipid and glucose abnormalities over a two-year period, comparable in time to our study. Neither we nor Rifas-Shiman could evaluate attempts at management, so reasons for increased documentation such as guideline adherence could not be assessed. Our analysis extends prior studies by evaluating whether those with documentation of risk factors had those risk factors in control according to Adult Treatment Panel III (ATP III) metabolic syndrome guidelines for a patient with average cardiovascular risk.

Molenaar *et al *studied rates of treatment and control of hypercholesterolemia, hypertension, and diabetes in normal weight, overweight, and obese subjects with a history of these conditions utilizing a CVD-free subset of the Framingham Heart Study. They found that subjects with hypercholesterolemia and hypertension who were obese were more likely to have these conditions treated than normal weight subjects. Rates of control of hypertension and hypercholesterolemia were uniformly poor and did not differ between weight groups. Rates of control of diabetes were poor among all three weight groups, but subjects who were obese were less likely to have control of fasting blood glucose than normal weight subjects. The goal of our study, however, was different from that of Molenaar. Molenaar studied rates of treatment and control of hypercholesterolemia, hypertension, and diabetes in normal weight, overweight, and obese subjects with a history of these conditions who were free of CVD, a subgroup of patients with a clear indication for BMI screening. Our goal was to evaluate in all patients, both with and without obesity-associated risk factors, the current state of BMI screening and subsequent screening for associated risk factors by BMI group, according to HEDIS and NHLBI guidelines. Molenaar *et al *examined a well-known, standardized study population, where only 196 subjects had missing BMI data. Our population was a non-standardized data source, and our subjects were a mixture of both those with and without CVD and its risk factors, with further subanalysis in patients with a history of CVD and diabetes. Despite these differences, findings from the structured Framingham population and from our analysis of usual clinical care are strikingly similar. This minimizes to a large degree the concern that high rates of missing BMI information could have distorted our findings, and may explain why our percentages of control were higher than those found by Molenaar, albeit still low overall [29].

The rapidly growing prevalence of obesity has pushed BMI assessment to appropriate prominence as a newly proposed reportable HEDIS measure. BMI assessment will be made by several means, including survey of EHRs in health care networks. Our results suggest that the HEDIS *BMI Assessment *measure has potential to provide a timely quality and safety foundation to improve care for patients with obesity. At least in our large primary care network, there clearly is substantial room for improvement in documentation of BMI and documentation and control of BMI-associated risk factors. While height and weight and BMI documentation may reflect individual physician practice styles, by speaking with medical directors we found that lack of height and weight appeared to be a clinic-level and not an individual PCP issue, with height and weight recording often performed or not performed before the PCP sees the patient. It is expected that introduction of the HEDIS *BMI Assessment *measure will improve this state of affairs, although the effectiveness of BMI documentation alone to improve care remains to be demonstrated.

According to our study, patients with already documented CVD or diabetes are more likely to have risk factor documentation and control regardless of BMI category. Those without a documented history of CVD or diabetes demonstrated more variation in risk factor documentation and control by BMI category. At least two recent studies corroborate these data. Melamed *et al *measured BMI in 289 patients in seven family practice clinics in Israel, and found that BMI was documented in only half of obese patients and 39% of overweight patients, and that patients with other chronic medical conditions were more likely to have BMI documented than those without documented comorbidities [30]. Waring *et al *looked at 2,330 overweight and obese patients included in the Cholesterol Education and Research Trial, and found increased odds of overweight or obesity management in relation to weight-related comorbidities for those with moderate or severe obesity [31].

Risk factor control appears to be related to a previously diagnosed risk factor and not to obesity. These findings become even more relevant in light of recent studies that demonstrate increased CVD risk in patients who are overweight and obese compared to normal weight patients, independent of hypertension and hypercholesterolemia [32]. This implies an important need to recognize overweight and obesity, ideally using a simple technique such as BMI, in order to enhance CVD and diabetes complications prevention. Our findings suggest that PCPs are aware of CVD, diabetes, and obesity as strongly tied risk factors, but that they may not recognize obesity as a risk factor for morbidity and mortality independent of these other comorbidities.

### Limitations

Our results must be interpreted with some limitations in mind. This is clinical, not research, data, which naturally suffers from information that is missing and inconsistent in its recording. We carefully addressed missingness with multiple methods of ascertaining exposures, and addressed inconsistencies in data recording by removal of clinically illogical extreme outliers. Evaluation of BMI documentation rates, with the inherent missing data, was a goal of our study. Furthermore, despite its limitations, evaluation of clinical data is a strength of this study, in that it provides a glimpse into current obesity care and insights into improving this practice. Although our data were derived from a single academic health care network, the sites included a representative mixture of urban, suburban, and hospital-based practices, making our findings generalizable and potentially replicable. Another important strength of our study is the use of a long-standing, widely used EHR encompassing all aspects of patient care from a large network of diverse clinics and patient populations. EHRs, while not a perfect tool due to the potential for improperly entered or overlooked data, have great potential for research, with studies showing that EHRs have potential for increasing documentation and treatment of obese patients [33]. Finally while there were differences seen in the percentages of documentation of risk factors, this may be due mainly to test indication, whereby certain tests such as cholesterol levels are more likely to be ordered on most patients than fasting glucose. However, our overall documentation numbers were large enough to yield consistent results across BMI categories.

## Conclusions

It is well-accepted that intentional weight loss mitigates many of the risk factors associated with obesity. Despite rising rates of obesity, physicians appear not to routinely assess BMI during office visits [34]. According to the NCQA, multiple organizations recommend measuring BMI as part of the routine physical examination [16,9,10,14,15]. Treatment recommendations for obesity depend on ascertainment of BMI and complications of obesity. Therefore, screening for BMI and comorbidities could change patient management. In light of new studies implicating overweight and obesity as independent risk factors for CVD, recognition of BMI becomes even more important in the primary care setting. We examined a large, diverse primary care network to evaluate current care and found that EHRs will be a useful tool to evaluate BMI assessment. We demonstrate substantial opportunities for improvement in the assessment and control of adiposity and associated risk factors that are needed to address the US obesity epidemic.

## Abbreviations

(ATP III): Adult Treatment Panel III; (BP): Blood Pressure; (BMI): Body Mass Index; (CVD): Cardiovascular disease; (CDC):Centers for Disease Control; (EHR): Electronic Health Record; (HEDIS): Healthcare Effectiveness Data and Information Set; (ICD-9): International Classification of Diseases, Ninth Revision; (LDL): Low-density Lipoprotein; (MGH): Massachusetts General Hospital; (NCQA): National Committee for Quality Assurance; (NHLBI): National Heart, Lung, and Blood Institute; (NIDDK): National Institute of Diabetes and Digestive and Kidney Diseases; (NW): Normal weight; (OB): Obese; (OW): Overweight; (PBRN): Practice-Based Research Network; (PCP): Primary Care Physician; (RPDR): Research Patient Data Repository; (US): United States.

## Competing interests

The authors declare that they have no competing interests.

## Authors' contributions

SR helped to conceive the study, developed the study design, performed the statistical analysis, and drafted the manuscript. AT assisted with data retrieval and performed the natural language processing abstraction. RG participated in the design of the study and assisted with data retrieval. JM helped to conceive the study design, assisted with data retrieval, assisted with the statistical analysis, and assisted with editing the manuscript. All authors read and approved the final manuscript.

## Pre-publication history

The pre-publication history for this paper can be accessed here:

http://www.biomedcentral.com/1472-6963/9/236/prepub

## References

[B1] Pi-Sunyer F Medical hazards of obesity Ann Intern Med 1993 119 7 pt 2 655 60 8363192 10.7326/0003-4819-119-7_part_2-199310011-00006

[B2] Diabetes Prevention Program Research Group Reduction in the incidence of type 2 diabetes with lifestyle intervention or metformin NEJM 2002 346 393 403 10.1056/NEJMoa012512 11832527 PMC1370926

[B3] Mehotra C Naimi T Serdula M Bolen J Pearson K Arthritis, body mass index, and professional advice to lose weight: implications for clinical medicine and public health Am J Prev Med 2004 27 1 16 21 10.1016/j.amepre.2004.03.007 15212770

[B4] Lopez-Jimenez F Malinski M Gutt M Sierra-Johnson J Aude Y Rimawi AA Mego PA Thomas RJ Allison TG Kirby B Hughes-Borst B Somers VK Recognition, diagnosis, and management of obesity after myocardial infarction International journal of obesity 2005 29 137 141 10.1038/sj.ijo.0802831 15520829

[B5] Buse J Ginsberg H Bakris G Clark N Costa F Eckel R Fonseca V Gerstein H Grundy S Nesto R Pignone M Plutzky J Porte D Redberg R Stitzel K Stone N Primary prevention of cardiovascular diseases in people with diabetes mellitus: a scientific statement from the American Heart Association and the American Diabetes Association Circulation 2007 115 114 126 10.1161/CIRCULATIONAHA.106.179294 17192512

[B6] Anderson J Kendall C Jenkins D Importance of weight management in type 2 diabetes: review with meta-analysis of clinical studies Journal of the American college of nutrition 2003 22 5 331 339 14559925 10.1080/07315724.2003.10719316

[B7] Douketis JD Feightner JW Attia J Feldman WF Canadian Task Force on Preventive Health Care Periodic health examination, 1999 update: 1. Detection, prevention and treatment of obesity CMAJ 1999 160 4 PMC1230077 10081468

[B8] Barlow SE Dietz WH Obesity evaluation and treatment: expert committee recommendations Pediatrics 1998 102 e29 10.1542/peds.102.3.e29 9724677

[B9] U.S. Preventive Services Task Force Screening for obesity in adults: recommendations and rationale Annals of internal medicine 2003 139 11 930 932 14644896 10.7326/0003-4819-139-11-200312020-00012

[B10] Executive summary of the clinical guidelines on the identification, evaluation, and treatment of overweight and obesity in adults 1998 http://www.ncbi.nlm.nih.gov/bookshelf/br.fcgi?book=obesity Accessed May 29, 2007 9810255

[B11] Screening and interventions to prevent obesity in adults 2003 http://www.ahrq.gov/clinic/USpstf/uspsobes.htm Accessed May 14, 2008

[B12] Nawaz H Katz DL American College of Preventive Medicine practice policy statement weight management counseling of overweight adults Am J Prev Med 2001 21 1 73 78 10.1016/S0749-3797(01)00317-8 11418263

[B13] Wylie-Rosett J Albright AA Apovian C Clark NG Delahanty L Franz MJ Hoogwerf B Kulkarni K Lichtenstein AH Mayer-Davis E Mooradian AD Wheeler M 2006-2007 American Diabetes Association nutrition recommendations: issues for practice translation Journal of the American Dietetic Association 2007 107 8 1296 1304 10.1016/j.jada.2007.05.009 17659893

[B14] Prevention and management of obesity (mature adolescents and adults) http://www.guideline.gov/summary/pdf.aspx?doc_id=10226&stat=1&string= Accessed May 14, 2008

[B15] Identification, evaluation, and treatment of overweight and obesity in the adult http://www.mqic.org/pdf/obesity_05.pdf Accessed May 14, 2008

[B16] Body Mass Index (BMI) Assessment for Adults (BAA) 2008 http://www.ncqa.org/Portals/0/PublicComment/HEDIS2009/BAA_Specs_and_Workup_PDF.pdf Accessed April 25, 2008

[B17] HEDIS 2009 Public Comment 2008 http://www.ncqa.org/tabid/661/Default.aspx Accessed July 30, 2008

[B18] Hivert MF Grant RW Shrader P Meigs JB Identifying primary care patients at risk for future diabetes and cardiovascular disease using electronic health records BMC Health Serv Res 2009 9 170 10.1186/1472-6963-9-170 19772639 PMC2753330

[B19] Turchin A Pendergrass ML Kohane IS DITTO - A tool for identification of patient cohorts from the text of physician notes in the electronic medical record Proc AMIA Symp 2005 744 8 16779139 PMC1560516

[B20] Turchin A Kolatkar NS Grant RW Makhni EC Pendergrass ML Einbinder JS Using regular expressions to abstract blood pressure and treatment intensification information from the text of physician notes JAMIA 2006 13 6 691 695 16929043 10.1197/jamia.M2078 PMC1656954

[B21] Defining Overweight and Obesity 2007 http://www.cdc.gov/nccdphp/dnpa/obesity/defining.htm Accessed 5/28/08

[B22] Metabolic Syndrome 2007 http://www.americanheart.org/presenter.jhtml?identifier=534 (Accessed 5/28/08)

[B23] ATP III Guidelines At-A-Glance Quick DeskReference 2001 http://www.nhlbi.nih.gov/guidelines/cholesterol/atglance.pdf Accessed 5/28/08

[B24] Meigs JB Cupples LA Wilson PW Parental transmission of type 2 diabetes: the Framingham Offspring Study Diabetes 2000 49 12 2201 7 10.2337/diabetes.49.12.2201 11118026

[B25] DeFaria Yeh D Freeman MW Meigs JB Grant RW Risk factors for coronary artery disease in patients with elevated high-density lipoprotein cholesterol American Journal of Cardiology 2007 99 1 1 4 10.1016/j.amjcard.2006.07.053 17196452

[B26] Grant RW Cagliero E Sullivan CM Dubey AK Estey GA Weil EM Gesmundo J Nathan DM Singer DE Chueh HC Meigs JB A controlled trial of population management: diabetes mellitus: putting evidence into practice (DM-PEP) Diabetes Care 2004 27 10 2299 2305 10.2337/diacare.27.10.2299 15451891

[B27] Lemay CA Cashman S Savageau J Fletcher K Kinney R Long-Middleton E Underdiagnosis of obesity at a community health center J Am Board Fam Pract 2003 16 1 14 21 10.3122/jabfm.16.1.14 12583646

[B28] Rifas-Shiman SL Forman JP Lane K Caspard H Gillman MW Diabetes and lipid screening among patients in primary care: a cohort study BMC health services research 2008 8 25 18234107 10.1186/1472-6963-8-25 PMC2266727

[B29] Molenaar EA Hwang S Vasan RS Grobbee DE Meigs JB D'Agostino RB Levy D Fox CS Burden and rates of treatment and control of cardiovascular disease risk factors in obesity: the Framingham heart study Diabetes care 2008 31 7 1367 72 10.2337/dc07-2413 18375414 PMC2453683

[B30] Melamed OC Nakar S Vinker S Suboptimal identification of obesity by family physicians Am J Manag Care 2009 15 9 619 24 19747026

[B31] Waring ME Roberts MB Parker DR Eaton CB Documentation and management of overweight and obesity in primary care J Am Board Fam Med 2009 22 5 544 52 10.3122/jabfm.2009.05.080173 19734401 PMC3967526

[B32] Bogers RP Bemelmans WJ Hoogenveen RT Boshuizen HC Woodward M Knekt P van Dam RM Hu FB Visscher TL Menotti A Thorpe RJ Jr Jamrozik K Calling S Strand BH Shipley MJ for the BMI-CHD Collaboration Investigators Association of overweight with increased risk of coronary heart disease partly independent of blood pressure and cholesterol levels: a meta-analysis of 21 cohort studies including more than 300,000 persons Arch Intern Med 2007 167 16 1720 8 10.1001/archinte.167.16.1720 17846390

[B33] Bordowitz R Morland K Reich D The use of an electronic medical record to improve documentation and treatment of obesity Fam Med 2007 39 4 274 9 17401772

[B34] Jackson J Doescher M Saver B Hart L Trends in professional advice to lose weight among obese adults J gen intern med 2005 20 814 8 10.1111/j.1525-1497.2005.0172.x 16117748 PMC1490207

